# A highly challenging balance training intervention for people with multiple sclerosis: a feasibility trial

**DOI:** 10.1186/s40814-023-01265-7

**Published:** 2023-03-15

**Authors:** A. Wallin, E. Franzén, U. Ekman, F. Piehl, S. Johansson

**Affiliations:** 1 Department of Neurobiology, Care Sciences and Society, Division of Physiotherapy, Karolinska Institutet, Huddinge, Sweden; 2 Rehab Station Stockholm, Research and Development Unit, Solna, Sweden; 3 Women’s Health and Allied Health Professionals Theme, Medical Unit Occupational Therapy and Physiotherapy, Karolinska University Hospital, Stockholm, Sweden; 4 Stockholm Sjukhem Foundation, R&D Unit, Stockholm, Sweden; 5 Department of Neurobiology, Care Sciences and Society, Division of Clinical Geriatrics, Karolinska Institutet, Stockholm, Sweden; 6 Women’s Health and Allied Health Professionals Theme, Medical Unit Medical Psychology, Karolinska University Hospital, Stockholm, Sweden; 7 Department of Clinical Neuroscience, Center for Molecular Medicine, Karolinska Institutet, Stockholm, Sweden; 8 Department of Neurology, Karolinska University Hospital and Neuroimmunology Unit, Stockholm, Sweden

**Keywords:** Balance control, Balance training, Dual-task, Feasibility, Highly challenging, Intervention development, Multiple sclerosis, Outcome response, Progression criteria, Stakeholder involvement

## Abstract

**Background:**

Balance training interventions with a gradual progression of difficulty and highly challenging tasks designed specifically for people with multiple sclerosis (MS) are rare. The objective was to adapt a balance training intervention originally developed for Parkinson’s disease through a co-design process and then conduct a pilot trial in MS to evaluate the feasibility of a large, full-scale study.

**Methods:**

Twelve people with MS with mild to moderate overall MS-disability were included in this single-group feasibility trial. Participants received one-hour training sessions twice or three times weekly for 10 weeks. The assessment included tests of physical and cognitive functioning and patient-reported quality of life-related outcomes. Data on feasibility aspects were collected at baseline and follow-up assessments and three times during the intervention period to inform the recruitment process, as well as to monitor retention and inclusion rates, study procedures, intervention delivery, and dynamic changes in the selected potential outcome measures. Progression criteria were used to determine whether to proceed to a full-scale trial. Descriptive statistics were used to present the data.

**Results:**

Out of six progression criteria, only retention and attendance at training sessions were not met. Reasons reported for not completing the intervention period mainly depended on external circumstances beyond the control of the study. In contrast, study procedures, intervention delivery, and intervention content (progression, adjustment, and control of challenge level of exercises) were considered feasible for a future, full-scale trial. The Mini-BESTest, which was used for the assessment of balance control, was considered suitable as the primary outcome in a full-scale trial with no ceiling or floor effects. Further, the Mini-BESTest showed a positive trend in outcome response with a median difference of 3.5 points between baseline and follow-up assessments. The power calculation performed suggests a feasible number of participants for recruitment.

**Conclusions:**

Overall trial aspects and intervention delivery were deemed feasible for a full-scale trial, but adjustments are needed to increase retention and attendance.

## Key messages regarding feasibility

- *What were the uncertainties regarding feasibility?* The uncertainties regarding feasibility were the recruitment process and rates of inclusion and retention; the study procedures; the intervention delivery; and the suitability of potential outcome measures.
- *What are the key feasibility findings?* The overall trial design and delivery of the intervention proved to be feasible. Four out of six progression criteria were met and the Mini-BESTest was considered suitable as the primary outcome.
- *What are the implications of the feasibility findings for the design of the main study?* The findings indicate that a full-scale trial is feasible; however, factors related to retention—how to facilitate participants' trial completion—and intervention delivery will be considered prior to a full-scale trial.

## Introduction

Multiple sclerosis (MS), a chronic inflammatory and neurodegenerative disease of the central nervous system mostly affecting women, is a leading cause of non-traumatic neurological disability among young and middle-aged adults [1, 2]. People with MS (PwMS) may display a wide range of symptoms including impairments in muscle strength and coordination, vestibular function, proprioception, vision, eye movement control, and cognition, as well as impaired integration of these functions [3]. Alone or in combination, these impairments often limit balance control, mobility and ambulation [4–6], even in the early stages of the disease [7].

Limited balance contributes to an increased fall risk, often coupled with a fear of falling [8, 9], and most frequently it occurs in people with mild to moderate MS disability before walking aids become a necessity [10]. Limitations in mobility and ambulation restrict the capacity for social participation and negatively impact health-related quality of life [11, 12]. Balance control requires the interaction between multiple underlying physiological systems, including biomechanical constraints, movement strategies, sensory strategies, orientation in space, control of dynamics, and cognitive processing skills [13]. In order to counteract balance limitation, interventions that aim to improve balance control should therefore include exercises that challenge these physiological systems. Intensity (i.e., level of challenge), in balance training has been defined as “the degree of challenge to the balance control system relative to the capacity of the individual to maintain balance” [14]. Further, to maximize the effectiveness of the training a high level of challenge should be maintained throughout an intervention period [15]. A number of studies aiming to improve balance in PwMS have been conducted, but variability in intervention types, outcome measures, and methodological limitations restrict the ability to draw more definitive conclusions on effectiveness [16]. However, several balance interventions have shown promising results [17–19], although methods for monitoring the level of challenge are rarely described in these studies.

A further limitation for understanding how a physical exercise intervention impacts clinical or patient reported outcomes is the lack of objective physiological biomarkers, for example, immunological markers in blood, although some progress has been made more recently [20, 21]. These preliminary observations, however, need to be replicated and extended in larger high-quality studies [22], and it remains to be shown if this also is relevant in a balance training intervention with a high level of challenge.

In sum, there is a paucity of balance training interventions specifically adapted for PwMS that utilize a gradual progression of difficulty and complexity in exercises and with a continuously controlled high level of challenge in exercises throughout the intervention period. Furthermore, since existing evidence is limited to the potential positive effects on balance control and fall risk over the short term, longer observation periods are needed to explore the durability of treatment effects [16]. However, prior to embarking on a resource intensive full-scale trial, careful consideration of feasibility aspects and potential outcomes is needed [23, 24]. Therefore, the objective of this pilot trial was to evaluate the feasibility aspects of a highly challenging balance intervention for PwMS.

## Methods

### Trial design

The present pilot study had a single-group feasibility trial design [25].

### Intervention development process

**Fig. 1:**
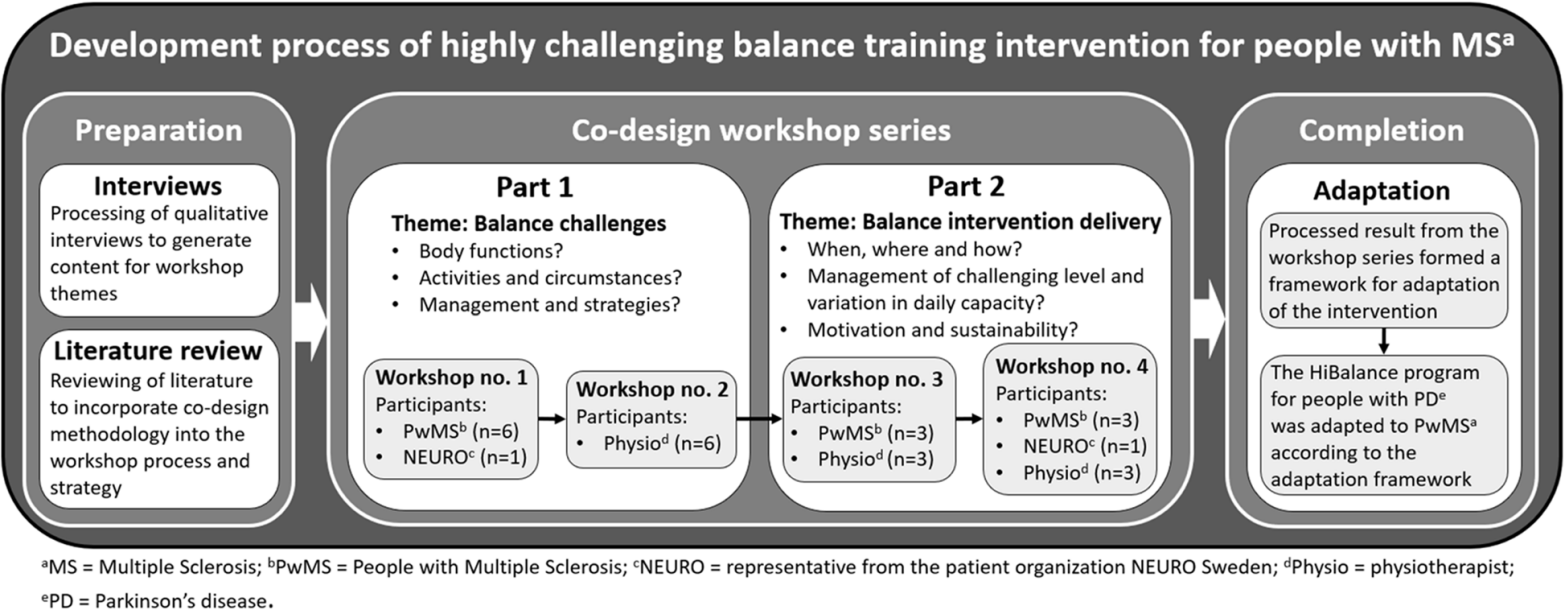
Overview of the co-design process for intervention development

The intervention was based on a highly challenging balance intervention delivered as a group training (the HiBalance program) in people with Parkinson´s disease, described elsewhere [26]. In brief, the HiBalance program consists of exercises that emphasize highly challenging aspects of balance control including cognitive and motor dual-tasks, which lead to significantly improved balance, gait velocity, step length and dual-tasking ability among people with Parkinson´s disease [27]. In order to adapt the program to PwMS, we employed a co-design process with a series of workshops (Fig. 1).

#### The co-design process

The co-design process consisted of a workshop series with the participation of different stakeholders. Eligible participants with MS were PwMS aged 18 to 65 years, with limited balance but who retained walking capacity indoors without walking aids. Six PwMS participated in the workshop series along with one representative from the patient organization NEURO Sweden and six physiotherapists specialising in MS rehabilitation. At each workshop, participants were selected to stimulate and support participant cooperation and discussion.

Participants could choose to participate in workshops face-to-face or via video-link. Four workshop sessions were conducted (Fig. 1), each lasting approximately three hours. The first two sessions (part 1) covered the theme *balance challenges;* and the final two sessions (part 2) covered the theme *balance intervention delivery.* Each participant participated once in each part (see Fig. 1). The results of the workshop series showed that among the participants, impaired motor-sensory function, stimuli-rich environments, cognitive processing, and awareness of capacity were factors that presented challenges in maintaining balance (part 1). In terms of the balance intervention delivery, participants reported control of variability in daily capacity and level of challenge in the intervention, as vital factors for the adequate individual adaptation of the exercises (part 2). The outcomes of the co-design process formed the framework for the adaptation of the HiBalance intervention to PwMS.

#### The intervention—highly challenging balance training for PwMS

**Fig. 2:**
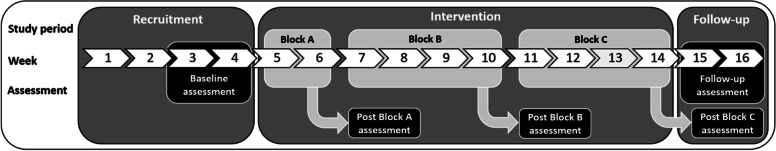
Planned timeline for the feasibility trial of a highly challenging balance training intervention for people with multiple sclerosis

The intervention delivered in the subsequent feasibility trial was an individually adjusted progressive group training aimed to challenge the subsystems for balance control, specifically stability limits, motor agility, anticipatory postural adjustments, and sensory integration. Over the intervention period, the level of difficulty and complexity in the balance exercises were increased in the consecutive blocks—A, B, and C (see Fig. 2). Block A included basic single-task exercises based on the balance control components. For progression in block B, a cognitive or a motor dual-task was added separately to the basic exercises. In block C, exercises and dual-tasks were combined for enhanced complexity. Exercises were individually adjusted, for example, by altering and changing base of support, gait speed, vision and/or adding dual-tasks. A framework for how the exercises within the above balance control components were designed is available from the corresponding author (A.W.).

Participants in the feasibility trial volunteered to participate in two or three weekly one-hour training sessions for 10 weeks. A general plan for the training sessions was set for the intervention period, with six to eight participants at each session. The details of the session content in the blocks were planned by one physiotherapist, who was present at each session. An additional four physiotherapists alternated as trainers. Of the five available trainers, two were present at each session.

At the start of each training session, participants rated their daily variation in capacity on a rating scale from 1 to 10, where 1 represented the worst imaginable capacity and 10 represented the best imaginable capacity. The session continued with a warm-up (≈ 10 min), balance exercises performed individually (≈ 25 min), and a group exercise with an obstacle course (≈ 20 min). At the end of each session (≈ 5 min), each participant rated the level of challenge of the training session on a rating scale. The scale ranged from 1 to 6 (1 = too low; 2 = low; 3 = fairly low; 4 = somewhat high; 5 = high; and 6 = too high). Reflections and feedback on how the level of challenge best could best be adjusted in the different exercises were given.

### Recruitment of participants and planned timeline for the feasibility trial

Inclusion criteria for the feasibility trial were PwMS diagnosed according to the 2017 revised McDonald criteria [28, 29], aged 18 to 65 years, and the ability to walk 100 m without aid. An additional inclusion criterion was an overall MS-disability score of 2.0 to 5.5 according to the Expanded Disability Status Scale (EDSS) [30], which quantifies the individual's overall disability through the assessment of eight functional systems. Criteria for exclusion were cognitive impairment as indicated by a score < 21 in the Montreal Cognitive Assessment (MoCA) [31], presence of other conditions that would substantially influence balance, an MS relapse or change of disease-modifying treatment within the past 8 weeks, alcoholism, or pregnancy.

Participants were recruited in August 2021 through an advertisement at MS specialist and clinical rehabilitation centers in Stockholm, Sweden, and through the patient organization NEURO Sweden. Potential participants were initially screened for eligibility by telephone before assessment for inclusion.

All included participants signed an informed consent; the study procedures were conducted in accordance with the Declaration of Helsinki. The ethical review board in Stockholm approved the trial (Nos. 2018/374-31, 2019-01562 and 2020-05952). The feasibility trial was conducted according to the timeline in Fig. 2, with a 4-week recruitment period, a 10-week intervention period, and a 2-week follow-up assessment period.

### Data collection

Data collection was carried out during 2-week assessment windows at baseline and at study completion. The assessments were conducted at the movement laboratory at Karolinska Institutet, Stockholm, Sweden, except for blood sampling, which was conducted at another location (the Academic Specialist Center, Stockholm Health Services, Stockholm, Sweden). Demographic information and information on fall frequency, use of mobility aids, education, years since MS diagnosis, and disease course were collected at baseline assessment through structured interviews. Data collection on feasibility aspects was collected throughout the study period, including structured interviews with participants at three time points during the intervention—post block A, post block B, and during the follow-up period, post block C. The data collection schedule for assessments of feasibility aspects and related uncertainties, including potential outcome measures tested for feasibility, is presented in Table 1.

**Table 1:** Data collection schedule on feasibility aspects and related uncertainties

Feasibility aspect	Assessments				
	Baseline	Post block			Follow-up
		A	B	C	
*Recruitment process and rates on inclusion and retention*					
Eligibility screening process (PC1)^a^ and inclusion rate (PC2)^a^	X				
Retention rate (PC3)^a^		X	X	X	X
*Study procedures*					
Information and communication accuracy				X	
Inclusion and exclusion assessment	X				
Assessment on demography and clinical characteristics	X				
Acceptability of time required for assessment procedures	X				X
Blood-sampling procedures				X	
Attitude towards long-term follow-up assessments				X	
*Intervention delivery*					
Training session time of day and weekly frequency				X	
Duration of training session				X	
Individual adjustment of:					
Exercises related to intervention progression		X	X	X	
Level of challenge across the progression blocks		X	X	X	
Exercises related to disability level and variation in daily capacity		X	X	X	
Monitoring of level of challenge		X	X	X	
Group training despite divergence in level of overall MS disability				X	
Individual balance exercises				X	
Safety in training				X	
Home exercise program as substitute for group training session				X	
Individual effort required for participation				X	
Motivation in training				X	
Attendance at training sessions (PC4)^a^				X	
Perceived intervention effect on balance control				X	
Willingness to recommend the training (PC5)^a^				X	
*Potential outcome measures*					
Primary outcome:					
Primary outcome suitability of the Mini-BESTest^c^ (PC6)^a^	X				X
Secondary outcomes:					
10-Meter Walk Test^c^	X				X
2-Minute Walk Test^c^	X				X
APDM^b^ Gait analysis (spatial and temporal gait parameters)	X				X
APDM^b^ Sway test	X				X
Six Spot Step Test^c^	X				X
Multiple Sclerosis Walking Scale^c^	X				X
Falls Efficacy Scale International^c^	X				X
Frenchay Activities Index	X				X
Multiple Sclerosis Impact Scale-29^c^	X				X
Modified Fatigue Impact Scale^c^	X				X
Hospital Anxiety and Depression Scale	X				X
EuroQol-5D (Index and Visual Analog Scale^c^)	X				X
Life Satisfaction Scale	X				X
Acceptance of Chronic Health Condition Scale	X				X
Trail Making Test^c^	X				X
Ray Auditory Verbal Learning Test^c^	X				X
Symbol Digit Modalities Test^c^	X				X
Immunological markers in blood	X				X

^a^*PC1 to PC6* progression criteria no. 1 to no. 6, see under heading "Progression criteria" in text

^b^*APDM* APDM’s Mobility Lab™ a body-worn sensor technology for assessment of balance and gait

^c^Trend in outcome response is reported in Results, Table 5

### Potential outcome measures

The Mini-Balance Evaluation Systems Test (the Mini-BESTest) [32, 33], an assessment of underlying physiological systems for balance control, was used as the potential primary outcome measure in a full-scale trial. To measure walking the 10-Meter Walk Test [34] with dynamic start and the 2-Minute Walk Test [35] were used. The Six Spot Step Test [36, 37] was used to measure complex walking. Spatial and temporal gait parameters and sway measures were assessed with APDM’s Mobility Lab™ [38].

To measure aspects of cognitive function, the Trail Making Test part B was used to assess cognitive flexibility by set-shifting [39], the Ray Auditory Verbal Learning Test to assess verbal episodic memory [40], and the Symbol Digit Modalities Test to assess cognitive processing speed [41, 42].

The 12-item Multiple Sclerosis Walking Scale [43] was used to assess the individual’s perceived impact of MS on walking ability, and the Falls Efficacy Scale International [44, 45] was used to assess the individual’s concerns about falling.

Further outcome measures used were: Frenchay Activities Index [46] for the frequency of social/lifestyle activities; Multiple Sclerosis Impact Scale-29 [47] for the physical and psychological impact of MS; Modified Fatigue Impact Scale [48, 49] for the impact of fatigue; Hospital Anxiety and Depression Scale [50, 51] for symptoms of depression and anxiety; EuroQol-5D (Index and Visual Analog Scale) [52] for health status; the Life Satisfaction checklist [53] for life satisfaction; and Acceptance of Chronic Health Conditions Scale [54–56] for acceptance of MS.

Feasibility in the blood sampling procedure was assessed. Blood-samples were collected in standard EDTA tubes for the level assessment of, for example, inflammatory markers in plasma. Blood samples were centrifuged (1500 G; 15 min) immediately after collection; the plasma was then separated, aliquoted and stored at − 80 °C until further analyses.

### Progression criteria

Six quantitative progression criteria (PC), were pre-defined to determine whether to proceed to a full-scale trial:

- PC1 ≥ 75% of interested PwMS were found eligible in the screening process
- PC2 ≥ 80% of eligible PwMS assessed for inclusion were included
- PC3 ≥ 80% of included participants were retained at follow-up
- PC4 ≥ 80% attendance at training sessions among included participants
- PC5 ≥ 80% of included participants would recommend the intervention to others
- PC6 ≤ 15% floor and ceiling effects in the Mini-BESTest

### Sample size and analytical methods

The sample size in the feasibility trial was set at 12 participants, divided into two training groups, in order to ensure safe balance training with high quality (progressive and individually adjusted) led by two trainers at each training session. Uncertainties regarding the relevant feasibility aspects were summarized and evaluated. Median, minimum, and maximum values; frequency; and percent were used to present quantitative data. Changes from baseline to follow-up were reported descriptively as trend directions in the outcome response. Change was analysed by calculating change in variables (follow-up value minus baseline-value) and expressed as median difference. Attendance at training sessions was calculated as the number of sessions attended divided by the number of sessions participants planned to attend. It was reported as a median value (1) for the whole sample and (2) for the participants that completed the follow-up assessment. IBM SPSS Statistics version 28 was used for statistical analysis.

A sample size calculation for a full-scale trial was performed, based on the difference between the intervention group and the control group for the Mini-BESTest. The difference (delta), was expected to be 3.3 points, based on the result from a similar study of balance training using the same outcome measure [17]. A two-sided *t* test was used with the level of significance set at .05, with 80% power, and with an assumption of a dropout of 20%. The calculations were performed using Stata version 15 (StataCorp, College Station, TX, USA).

## Results

### Recruitment process and rates of inclusion and retention

**Fig. 3:**
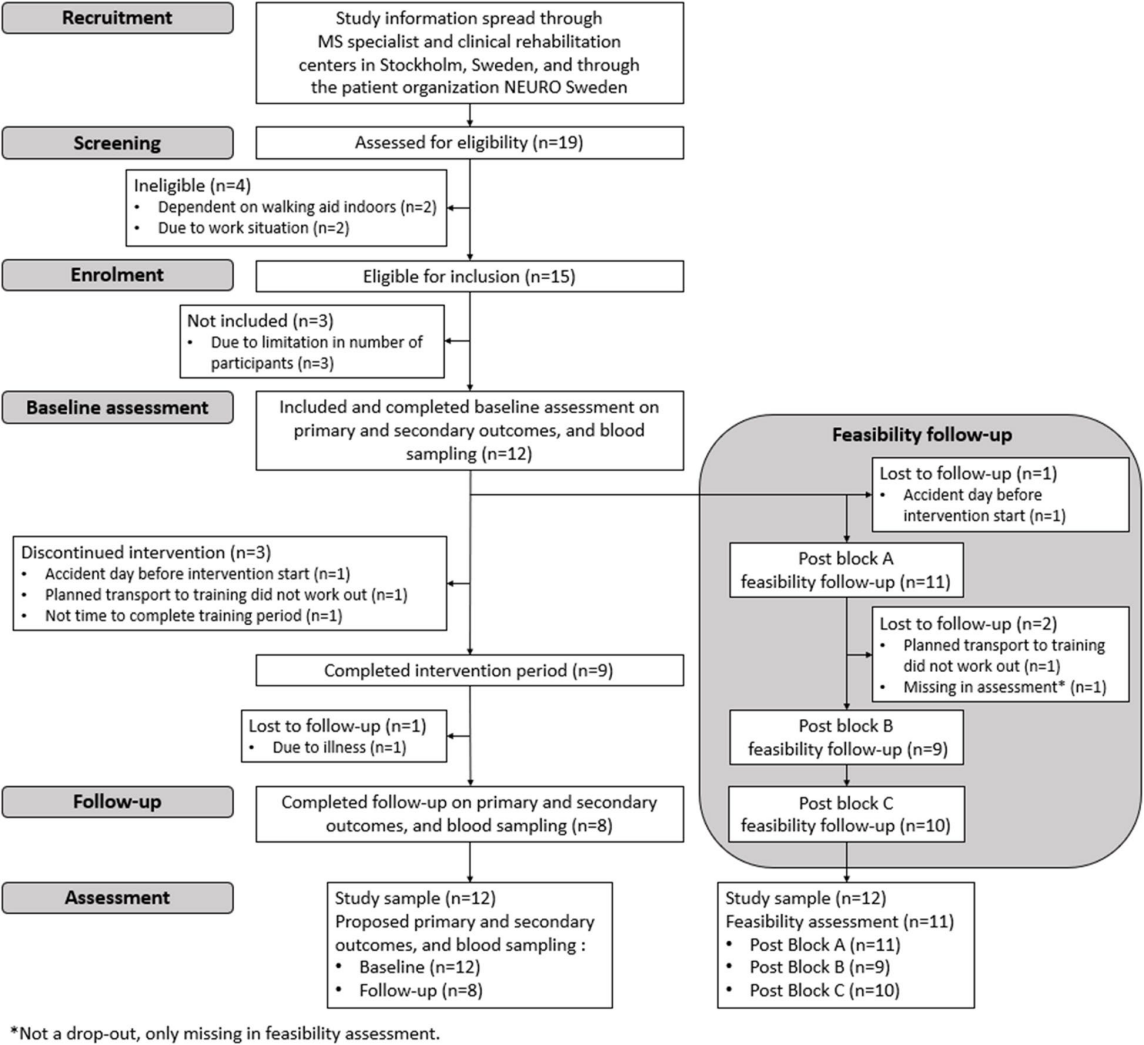
Consort flow diagram of participants

Participant flow from recruitment to follow-up assessment is presented in Fig. 3. Nineteen PwMS expressed interest in participation in the intervention. Of those, 15 PwMS (79%) were eligible, which met the requirement for PC1 (Table 2). The first twelve eligible PwMS who underwent inclusion assessments were included (100%), which met the requirement for PC2 (Table 2). Three eligible PwMS were not assessed for inclusion since the sample size was set at 12 participants. The demographics of the 12 included participants are presented in Table 3.

**Table 2:** Summary of results of progression criteria feasibility assessment

Progression criteria (PC)	Result	Criteria met or not	Course of action
PC1. Screening process for assessment of eligibility.	79% (*n* = 15) of interested PwMS were considered eligible after the screening process. *Calculations made on 19 PwMS.*	Met	Proceed
PC2. Feasibility to include participants to the trial.	100% (*n* = 12) of eligible PwMS who underwent inclusion assessments were included. *Calculations made on 12 PwMS.*	Met	Proceed
PC3. Feasibility to include enough participants to the trial, with appropriate retention to follow-up.	67% (*n* = 8) of included participants were retained at follow-up. *Calculations made on 12 PwMS.*	Not met	Considerations will be made whether it will be feasible to proceed.
PC4. Attendance at training sessions.	70% median attendance at training sessions during the intervention period. *Calculations made on 12 PwMS, of which 9 planned to participate in 2 and 3 to participate in 3 sessions per week.* 89% median attendance at training sessions during the intervention period. *Calculations made on 8 PwMS, of which 6 planned to participate in 2 and 2 to participate in 3 sessions per week.*^a^	Not met	Changes in the intervention set-up will be considered.
PC5. Willingness to recommend the intervention to others.	100% (*n* = 10) of participants reported willingness to recommend the intervention. *Calculations made on 10 PwMS.*	Met	Proceed
PC6. Suitability of the Mini-BESTest as the primary outcome for the targeted patient group.	8% (*n* = 1) of the included participants achieved the highest score and 0% (*n* = 0) the lowest score in the Mini-BESTest. *Calculations made on 12 PwMS.*	Met	Proceed

^a^Presentation of median attendance among 8 participants who completed the intervention and the follow-up assessment

**Table 3:** Demographic characteristics

Characteristics	People with MS^a^ (*n* = 12)
Sex, no. (%)	
Women	9 (75)
Men	3 (25)
Age, years, median (min-max)	54 (37–63)
Education, years, median (min-max)	15 (10–17)
Body mass index, kg/m^2^, median (min-max)	23.8 (18.9–33.5)
Have fallen last 6 months, no. (%)	
No	8 (66.7)
Yes	4 (33.3)
Use of walking aid, no. (%)	
No	6 (50)
Yes, outdoors^b^	6 (50)
Cognitive function, MoCA^c^, median (min-max)	26 (23–29)
Mild cognitive impairment according to MoCA^c^ (< 26), no. (%)	
No	7 (58.3)
Yes	5 (41.7)
Overall MS disability, EDSS^d^, median (min-max)	3.5 (2.5–5.5)
Mild impairment in ≥ two functional systems^e^, no. (%)	12 (100)
Moderate impairment in ≥ two functional systems^e^, no. (%)	6 (50)
Disease course, no (%)	
Relapsing remitting	9 (75)
Progressive	3 (25)
Years since diagnosis, median (min-max)	10 (2–24)

^a^*MS* multiple sclerosis

^b^Type of walking aid: unilateral (*n* = 4), bilateral (*n* = 2)

^c^*MoCA* Montreal Cognitive Assessment

^d^*EDSS* Expanded Disability Status Scale

^e^A total of eight functional systems are assessed in the EDSS: pyramidal, cerebellar, brainstem, cerebral, and visual functions, and further, bowel and bladder function, and ambulation

The completion of the intervention and the follow-up assessment were accomplished in eight participants, which resulted in a 67% retention rate, thereby failing to meet the PC3 criterion (Table 2). Three participants discontinued the intervention due to: trauma caused by an accident the day before intervention start (*n* = 1); malfunctioning transportation service (*n* = 1); and lack of time (*n* = 1). Furthermore, one participant could not be assessed within the 2-week follow-up window post-intervention due to illness.

### Study procedures

Post-intervention, participants reported that the preparatory information about the trial distributed during recruitment was consistent with their experiences of the intervention and study procedures (Table 4). Inclusion and exclusion assessments were considered feasible, as were the time required at baseline and follow-up assessments, and the blood-sampling carried out at a different location. Baseline and follow-up assessments lasted between 110 and 160 min, and 60 and 100 min, respectively (time for blood-sampling not included). Furthermore, the participants’ attitudes towards participation in long-term follow-up assessments in a future trial were positive (Table 4).

**Table 4:** Results on feasibility aspects and related uncertainties

Feasibility aspect	Summary of responses	Consideration
*Study procedures*		
Accuracy of information and communication about intervention and study procedures	All participants reported that information and communication corresponded with their experiences of the intervention and study procedures or that they had been pleasantly surprised about the difficulty and challenge level of the exercises in the intervention.	Feasible
Inclusion and exclusion assessment	The inclusion assessment with the Expanded Disability Status Scale and the exclusion assessment with the Montreal Cognitive Assessment were considered feasible.	Feasible
Assessment of demographic and clinical characteristics	Baseline data collection through semi-structured interview was considered feasible.	Feasible
Acceptability of time required for assessment procedures	Time required for the baseline and follow-up assessments at Karolinska Institutet were considered acceptable.	Feasible
Blood-sampling procedure	Sampling of blood was feasible.	Feasible
Attitude towards long-term follow-up assessments	All participants reported that they are likely to participate in long-term follow-up measurements up to 6 months post-intervention.	Feasible
*Intervention delivery*		
Training session time of day and weekly frequency	Several training sessions available, enabling the participants to choose session time and weekly frequency themselves, contributed to feasibility.	Feasible
Duration of training session	Training sessions of 60 min were feasible for most participants since the time spent on transport also needed to be considered. A few expressed a wish for longer sessions.	Feasible
Individual adjustment of exercises related to intervention progression	Initially, more careful guidance on how exercises could be adapted was required but participants gradually became more confident in adjusting the exercises themselves.	Feasible
Individual adjustment of challenge level across the progression blocks	The progression blocks were considered supportive to stimulate progression and create new challenges. The participants could gradually, through increased awareness and knowledge of their own ability, adjust progression and challenge level themselves. Guidance by the trainers was required to calibrate the exercise complexity and to suggest rest when needed.	Feasible
Individual adjustment of exercises related to disability level and variation in daily capacity	Initially, some participants reported the exercise challenge level to be somewhat low in relation to their own disability level, but the appropriate challenge level could subsequently be implemented. Individual adjustments related to variation in daily capacity were considered throughout the intervention period, which made exercises easier or more difficult, i.e., the challenge level was altered.	Feasible
Monitoring of level of challenge	The rating scale for assessment of challenge level in the balance training was reported to be comprehensible and easy-to-use. It was perceived easier to rate specific exercises than to make an overall assessment of the challenge level for an entire balance training session.	Feasible
Group training despite divergence in level of overall MS disability	Variations in overall MS disability between participants was perceived as a source of inspiration rather than a disadvantage as it contributed to reflection and new perspectives.	Feasible
Individual balance exercises	The individual balance exercises were considered relevant and useful where individual adjustment of challenge level specifically related to individual disabilities could be in focus.	Feasible
Safety in training	The intervention was perceived to be safe. Falling was considered a part of the risk when participating in a highly challenging balance intervention.	Feasible
Home exercise program as substitute for group training session	Training at home was considered a possible supplement, but could not replace guided group training sessions; the high intensity and challenge level carried out during training sessions could not be reached at home, and exercise equipment was not available.	Feasible but not requested
Individual effort required for participation	A majority of the participants stated a need to refrain from other activities (e.g., rearrangement of work and family activities) in order to be able to prioritize the intervention, since their regular schedule was full.	Feasible
Motivation in training	All participants reported feeling motivated to participate in the intervention. Factors contributing to motivation were the increased awareness and knowledge of their own balance capacity, which was accomplished through feedback given by the trainers and peer participants.	Feasible
Perceived intervention effect on balance control	Perceived effects of the intervention were increased safety and improved balance control or maintained balance control if an ongoing deterioration in physical status was present. Examples of enhanced confidence were climbing stairs without a handrail or walking on slippery or uneven surfaces.	Feasible
*Potential outcome measures*		
Assessment of primary and secondary outcomes	Mini-BESTest^a^	Feasible
	10-meter walk test^a^	
	2-minute walk test^a^	
	APDM^b^ Gait analysis (spatial and temporal gait parameters)	
	APDM^b^ Sway test	
	Six Spot Step Test^a^	
	Multiple Sclerosis Walking Scale^a^	
	Falls Efficacy Scale International^a^	
	Frenchay Activities Index	
	Multiple Sclerosis Impact Scale-29^a^	
	Modified Fatigue Impact Scale^a^	
	Hospital Anxiety and Depression Scale	
	EuroQol 5D (Index and Visual Analog Scale^a^)	
	Life Satisfaction Scale	
	Acceptance of Chronic Health Condition Scale	
	Trail Making Test^a^	
	Ray Auditory Verbal Learning Test^a^	
	Symbol Digit Modalities Test^a^	
	Immunological markers in blood	

^a^Trend in outcome response presented in Table 5

^b^*APDM* APDM’s Mobility Lab™ a body-worn sensor technology for assessment of balance and gait

### Intervention delivery

#### Time of day, frequency, and duration of training session

Participants reported that due to the opportunity to choose from various session times from several time slots every week, which participants were able to book themselves, the intervention was feasible (Table 4). Also, the duration of the sessions (1 h) was feasible for most participants. A few participants reported that they had the capacity to participate in even longer sessions, but that the time spent on transport to and from the training facility needed to be considered (Table 4).

#### Exercise progression and level of challenge adjustment

Participants reported that the exercise progression and level of challenge related to the individual adjustment of exercises across the three progression blocks was feasible (Table 4). The blocks (A, B, and C) created new challenges and stimulated progression. Initially, the participants expressed a need for more guidance on how exercises could be adapted, but they gradually grew more autonomous in making these adjustments. The participants experienced that the intervention increased awareness and knowledge of their own abilities. Some participants required guidance from the trainers to calibrate the exercise complexity and to prompt them to take time for recovery when needed. The trainers also reported that the adjustment of the progression of difficulty and level of challenge for the different participants was feasible (Table 4). Furthermore, individual adjustments were made with consideration for daily variation in capacity through both increased and decreased level of difficulty, which was considered feasible by both participants and trainers (Table 4). Initially, some participants reported that the level of challenge in the exercises was somewhat low in relation to their own individual disability level, but the level of challenge was then adjusted to an appropriate level (Table 4).

#### Monitoring the level of challenge

The rating scale for level of challenge in the balance training intervention was reported to be easy to understand and use and deemed relevant by both participants and trainers (Table 4). The rating of specific exercises performed during a session was also perceived to be easy, but rating the level of challenge for an entire training session was reported to be more difficult, since the level of challenge in various exercises could vary significantly, which also was confirmed by the trainers (Table 4).

#### Group training, individual exercises, and safety in training

Overall MS-disability differed among the participants, which was perceived in the group training as a source of inspiration rather than a disadvantage (Table 4). Inter-individual differences contributed to the participants’ reflection and new perspectives on their own balance. However, the part of the session part where exercises were performed individually was considered relevant and useful, as a greater focus was given to the adjustment of the level of challenge in relation to inter-individual differences. Furthermore, the intervention was perceived as safe by the participants. Three fall incidents occurred across three participants, none resulting in injury (Table 4). However, participants considered the fall risk to be an inevitable component of a highly challenging balance training program (Table 4).

#### Attitudes towards using a specified home exercise program

The intervention was delivered as a guided group training. Post-intervention, the participants were asked about their attitudes towards carrying out the training individually at home (Table 4). Participants responded that a home exercise program could serve as a complement to, but not a substitute for, the group training. The participants described the supervised group training as a necessity for attaining and maintaining a high level of challenge in the exercises, and this was not perceived to be possible exclusively in a home-setting (Table 4).

#### Individual effort required for participation and motivation

Even though most participants needed to rearrange work and family activities to fit the intervention into their weekly schedule, participation in the intervention was perceived as worth the effort (Table 4). Additionally, participants reported feeling motivated to participate in the intervention and that it contributed to improved awareness and knowledge of their own balance control (Table 4).

#### Attendance at training sessions

The median attendance at the sessions for the whole sample (i.e., all participants who planned to participate in either two or three training sessions per week) was 70% (PC4 not met, Table 2). However, the median attendance for the eight participants that completed the follow-up assessment was 89% (Table 2).

#### Perceived effect and willingness to recommend training

The majority of participants reported increased safety and improved balance control post-intervention. One participant described the effect as an experience of maintained balance control, despite the feeling that the level of disability continued to increase (Table 4). Additionally, all ten participants (100%) who participated in the post block C assessment of feasibility aspects reported a willingness to recommend the intervention to others for a future trial, which met the requirement for PC5 (Table 2).

### Outcome measures

#### Primary outcome

**Fig. 4:**
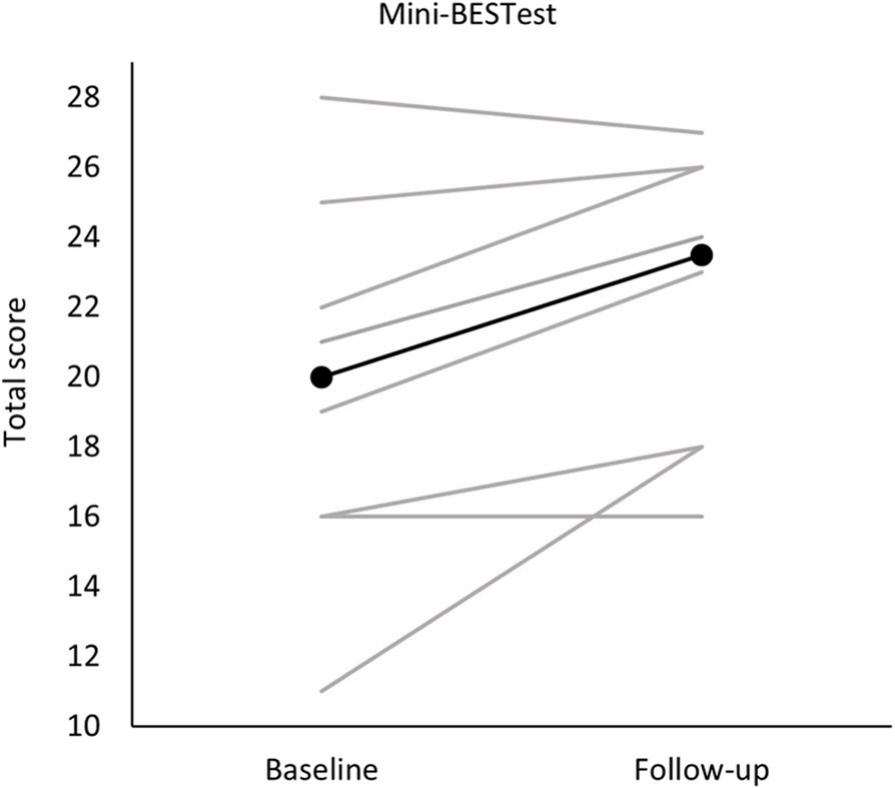
Trend in outcome response of the Mini-BESTest. Absolute values of the Mini-BESTest total score at baseline and follow-up assessment are presented with grey lines for individual participants and the group median values with a black line

The Mini-BESTest was suitable for the assessment of balance control in all participants. No floor or ceiling effect was found (ceiling effect 8%), which met the requirement for PC6 (Table 2). Furthermore, there was a positive effect trend gauged by the Mini-BESTest with a median difference between baseline and follow-up assessments of 3.5 points (see Table 5 and Fig. 4).

**Table 5:** Trend in outcome response on balance and walking

Outcome measure	Median difference (minimum to maximum) between baseline and follow-up	Direction^a^
Mini-BESTest^b^	3.5 (− 1 to 7)	+
10-Meter Walk Test, maximum gait speed (m/s)^b^	0.04 (− 0.04 to 0.25)	+
10-Meter Walk Test, self-selected gait speed (m/s)^b^	− 0.04 (− 0.17 to 0.23)	−
2-minute walk test, self-selected gait speed (m)^b^	− 10.2 (− 14.4 to 8.4)	−
Six Spot Step Test (s)^c^	− 1.7 (− 3.5 to 3.5)	+
Multiple Sclerosis Walking Scale-12 (Total score)^c^	− 5.5 (− 16 to 1)	+
Falls Efficacy Scale-International (Total score)^c^	1.5 (− 8 to 7)	−

^a^Direction indicates whether trend in change from baseline to follow-up assessment was positive (+), negative (−), or unchanged (0)

^b^Positive values indicate trend to improve; ^c^Negative values indicate trend to improve

#### Secondary outcomes

All secondary outcome measures, including the blood sampling procedure performed at another location, could be accomplished by all participants attending at baseline and follow-up assessments. The 10-Meter Walk Test showed a positive trend, with an increase in maximum gait speed, indicating walking improvement (Table 5). On the other hand, there was a slightly negative trend for the same test with self-selected gait speed. A similar negative trend was seen in the 2-Minute Walk Test, with a shorter distance covered at the follow-up assessment. The trend for the Six Spot Step Test was positive, indicating improvement in complex walking. The Multiple Sclerosis Walking Scale indicated improvement in the way participants perceived the impact of MS on their walking ability. However, the Falls Efficacy Scale International I showed a trend indicating increased concerns about falling (Table 5).

#### Sample size in at future full-scale trial

The sample size calculation, with the level of significance set at .05, with 80% power and with an assumption of a dropout of 20%, suggested to recruit 70 PwMS (35 in each group).

## Discussion

In this pilot study of a highly challenging balance intervention adapted for PwMS, we found that most feasibility aspects of the study procedures and intervention delivery were feasible, although some further adaptations should be considered to improve retention and training session attendance before embarking on a full-scale trial.

The representation of selected stakeholders in the co-design process promoted anchoring and acceptance among both providers and receivers of the intervention. Through the co-design process awareness and control of variability in capacity emerged as important factors for the adaptation of the HiBalance program to PwMS. Furthermore, continuously monitoring the level of challenge in the exercises also emerged as an important aspect, a finding in line with recent suggestions for maximization of the effectiveness of balance interventions [15]. These factors were therefore integrated into the intervention, emphasizing that the developed intervention is designed to meet the specific needs of PwMS.

Although the program was generally deemed feasible, two of six quantitative pre-defined PC for the feasibility assessment were not met. Both criteria, retention, and attendance at training sessions, were related to the degree of trial participation. Four participants did not complete either the intervention or both the intervention and the follow-up assessment which contributed to the low retention rate. External circumstances beyond the control of the study caused three of the dropouts, and even though some reasons for dropout were pandemic related, certain actions need to be considered when recruiting for a full-scale trial to prevent dropout. The fourth dropout was related to practical circumstances, where a shorter commute time to the training facility or a shorter intervention period could have facilitated completion. Regarding attendance, the criterion was not met for the whole sample but was met for those who completed the follow-up assessment. This implies that low retention negatively impacted the level of attendance at training sessions for the whole sample.

Most participants did not perceive participation in the intervention as particularly burdensome. During the training sessions, participants were able to adjust according to their daily capacity; on the other hand, participants reported that it was stimulating to try to reach a high level of challenge in the different exercises. The time required for baseline and follow-up assessments was considered acceptable. However, individual adjustments in assessment procedures were needed, such as short breaks between different tests, since participants had a varied level of capacity to perform the physical tests and other demanding parts of the intervention. Some participants perceived the physical assessments to be more energy intensive than others, while some reported that the more mentally demanding parts of the assessments were more exhaustive. The blood sampling procedure, which were carried out at another location, were considered feasible.

Assessments for inclusion or exclusion were primarily found to be feasible. Some difficulty occurred if the overall MS-disability level was low. In the lower range of the EDSS, the functional impairments are minimal or small, making the assessment more difficult. However, all 12 participants included were mildly impaired in at least two functional systems, and 6 of the 12 participants were moderately impaired in at least two functional systems (Table 3). The presence of impairments in several functional systems, which was also observed in people with a low EDSS score, reveals the complexity of cases where interacting impairments contribute to limited balance control in PwMS. To specifically target PwMS with balance limitations in a future full-scale trial, a measurement of balance control should be added to define balance limitations at inclusion, for example the Mini-BESTest, with an upper limit set for inclusion. Similar cut-off scores on outcomes have been set in other balance interventions in order to ensure higher precision in the criteria for reaching the targeted patient group [17, 57].

The flexibility in choosing and booking weekly sessions facilitated attendance, but is also more demanding from an organizational perspective. It remains to be shown how frequency and the overall length of the intervention may impact the feasibility of participation as well as the probability of attaining beneficial treatment effects. However, we deem it likely that this type of intervention should be delivered twice weekly for 10 weeks to be effective, based on the median attendance of 89% at training sessions during the intervention period among the eight participants who completed the follow-up assessment.

In the opinion of the participants, a home exercise program is not a suitable substitute for the supervised group sessions, since it is much less likely that they would attain the necessary level of difficulty in the exercises at home. However, home training could be a complement to supervised sessions, including core stability and fitness exercises, which are exercises that have previously been shown to positively impact balance control [18].

The participants reported that the intervention was safe, motivating, and contributed to an awareness and knowledge of their own balance control. After the intervention, they also reported increased safety and improved or maintained balance control in their everyday lives, which is in line with the trend found in follow-up assessment with the Mini-BESTest. Further, they reported that they would recommend the intervention to peers. Taken together, these findings lead to a positive initial assessment of the intervention and suggest that it should be tested in a full-scale trial.

Gunn et al. (2015) [16] concluded that supporting participants to reach an appropriate intensity in highly challenging balance exercises is critical to maximise intervention effectiveness. Methods aiming to control the level of challenge in balance exercises have been investigated in recent studies [17, 58]. However, the methods reported differ widely, from an overall ambition to increase the challenge level [58] to detailed instructions to the therapist on how to actively aim for a certain difficulty level [17]. Thus, as part of the development of the intervention in this study, we also developed a rating scale for the assessment of level of challenge in the training, in which the participant was encouraged to aim for a *fairly low* to *somewhat high* level of challenge. The participants perceived the rating scale as easy to understand and use for individual exercises, but found it more difficult to use when grading a whole session, which may need to be considered when designing future trials. Further, a validity and reliability evaluation of the scale is needed in future studies.

The built-in gradual exercise progression delivered in blocks, was considered an effective way to stimulate progression and to create new challenges throughout the intervention period. Initially, coaching was needed in the exercises, but participants gradually became more confident in adjusting the level of challenge in the exercises themselves. We consider this aspect important for the participants' experience that in addition to improved balance control, the intervention increased safety and self-confidence when moving around.

The Mini-BESTest was both feasible and suitable as the primary outcome for the targeted patient group. A positive trend in effect was seen as well as a median change after the intervention period, which was close to the minimal detectable change for the target group (i.e., 4 points) [33]. Furthermore, the results on maximum gait speed (10-Meter Walk Test), complex walking (Six Spot Step Test), and self-perceived impact of MS on walking ability (Multiple Sclerosis Walking Scale) pointed in the same direction. These results also aligned with the participants’ own perception of the intervention effect. In contrast, a negative trend was evident for self-selected gait speed. It can be speculated that this finding may depends on the increased awareness among participants of their balance limitations, but this may also depend on the fact that tests of walking performed with a lower gait speed tend to have greater variance, resulting in larger measurement error [59]. It would be of interest in a future full-scale trial to analyse the intervention effect on other qualitative aspects of walking apart from gait speed and distance covered, for example, stride length and step time. In addition, a test that evaluates dual-task capability related to balance control and gait should be included in a full-scale trial in the test procedure.

At baseline and follow-up assessments in the present trial, questionnaires were used to assess different aspects of health, disease impact, and capacity to be active. Due to the scope and duration of the trial, these outcome measures could not be reliably interpreted here, but it would be of interest for a future full-scale trial to see if there is an impact on quality of life measures at longer follow-up.

The sample size calculation suggested to recruit 70 PwMS (35 in each group). This sample size is considered a feasible number of participants to recruit and in line with similar interventions conducted within PwMS [17, 18].

### Strengths and limitations

A major strength of this feasibility trial was that a previously designed highly challenging balance intervention was, with stakeholder involvement, reused and developed to be applied to PwMS. Further, among a range of feasibility aspects most progression criteria for embarking on a full-scale trial were fulfilled. An additional strength is that the trial was conducted under similar conditions as a full-scale trial. In contrast, the main limitations comprise were the small sample size, as well as the lack of a randomization procedure, blinding, and a control group.

## Conclusions

In this pilot study, we evaluated a number of feasibility aspects important for a full-scale study, of which most feasibility aspects were met. However, adaptations to improve retention should be considered by taking dropouts into account when recruiting. The power calculation performed suggests a feasible number of participants to recruit. The result has relevance and applicability for a future full-scale trial of the highly challenging balance intervention specific to PwMS.

### Registration

Feasibility trial not registered.

### Protocol

No protocol published.

## Data Availability

With respect to Swedish and EU personal data legislation (GDPR), the data are not freely accessible due to regulations regarding personal privacy in research and public access. The data are available from the principal investigator of the project: Sverker Johansson (sverker.johansson@ki.se), on a reasonable request. Any sharing of data will be regulated via a data transfer and user agreement with the recipient.

## Abbreviations

MS: Multiple sclerosis
PwMS: People with multiple sclerosis
EDSS: Expanded Disability Status Scale
MoCA: Montreal Cognitive Assessment
PC: Progression criteria
Mini-BESTest: Mini-Balance Evaluation Systems Test
